# Progress in the application of isothermal amplification technology in the diagnosis of infectious diseases

**DOI:** 10.3389/fmicb.2025.1601644

**Published:** 2025-07-04

**Authors:** Qingshang Bi, Mengru Liu, Li Yan, Jun Cheng, Qingyang Sun, Yuzhu Dai, Lingli Zou

**Affiliations:** ^1^Department of Clinical Laboratory, Third Sanatorium, Air Force Healthcare Center for Special Services, Hangzhou, China; ^2^School of Laboratory Medicine, Bengbu Medical University, Bengbu, China; ^3^Department of Clinical Laboratory, The 903rd Hospital of The People’s Liberation Army, Zhejiang, China; ^4^Department of Health Economic, The 903rd Hospital of The People’s Liberation Army, Zhejiang, China

**Keywords:** isothermal amplification technology, infectious diseases, CRISPR/Cas system, molecular diagnostics, nucleic acid amplification

## Abstract

Rapid detection of infectious diseases is critical for global public health prevention and control. However, the use of traditional molecular diagnostic methods, including PCR, has been limited because of their cumbersome procedures, complex equipment requirements, operation at different temperatures, and the level of expertise required for operation. Isothermal amplification technology (IAT) provides a rapid, sensitive, specific, simple and less costly method for diagnosing infectious diseases, which has led to revolutionary breakthroughs in molecular diagnostics. This paper summarizes recent progress in IAT technology, which focuses on the principles and applications of core technologies such as NASBA, LAMP, RPA, and RAA. In addition, the combination of IATs with the CRISPR/Cas system, which further revolutionizes nucleic acid detection technology, is explored in this review.

## 1 Introduction

Infectious diseases, as defined as pathological disorders caused by invasive pathogenic microorganisms including bacteria, viruses, fungi, and parasites, continue to pose significant threats to global public health. These conditions are clinically distinguished from noncommunicable diseases by their transmissible nature and external pathogenic origin. Globally, infectious disease remains a leading cause of death and disability and a growing challenge to health security and human progress (Nii-Trebi, 2017). According to the World Health Organization (WHO), 13 million people died from communicable diseases in 2021, and people in low- and middle-income countries are far more likely to die from communicable diseases than from noncommunicable diseases (WHO, 2021). Rapid diagnosis of infectious diseases is critical to stop their spread. Although traditional molecular diagnostic techniques (such as PCR) are highly sensitive, their dependence on thermal cyclers, specialized laboratories, and skilled operators severely hinders their application in remote areas and during outbreaks (Obande and Banga Singh, 2020).

As a revolutionary breakthrough in molecular diagnostics, IAT overcomes the numerous limitations of traditional PCR. It enables rapid, portable detection of infectious diseases through an isothermal reaction process, delivers fast results, has high efficiency, and is compatible with simple equipment (Wang et al., 2023). Nucleic acid sequence-based amplification (NASBA) for rapid diagnostic detection of pathogenic viruses from RNA genomes was introduced in 1991 (Compton, 1991). In 2000, Notomi et al. developed loop-mediated isothermal amplification (LAMP), which has gained prominence because of its high specificity and rapidity (Notomi et al., 2000). Recombinase polymerase amplification (RPA), published in 2006 and commercialized by TwistDx, has emerged as a leading IAT (Piepenburg et al., 2006). A Chinese research team subsequently developed recombinase-aided amplification (RAA) based on its technical principles (Munawar, 2022). RAA has been used to optimize enzyme systems, control costs, and enhance local adaptability, offering unique advantages in emergency response scenarios such as SARS-CoV-2 screening and primary care. With the continuous improvement of IATs, combinations with other detection technologies, such as the CRISPR/Cas system, have been developed. Among the CRISPR/Cas associated proteins, the Cas12 and Cas13 proteins have been widely used for DNA and RNA detection (Guk et al., 2023). Mature and stable methods, such as SHERLOCK (Kellner et al., 2019) and DETECTR (Chen et al., 2018; Kellner et al., 2019), when combined with IAT, offer more versatile, portable and cost-effective solutions for infectious disease detection. This paper reviews recent advancements in IATs, focusing on NASBA, LAMP, RPA, RAA, and CRISPR/Cas-integrated IATs. Its core mechanism, technical characteristics, research progress, and current limitations are discussed, with the aim of providing theoretical support for accurate infectious disease diagnosis and public health prevention strategies.

## 2 Nucleic acid sequence-based amplification

**Figure 1:**
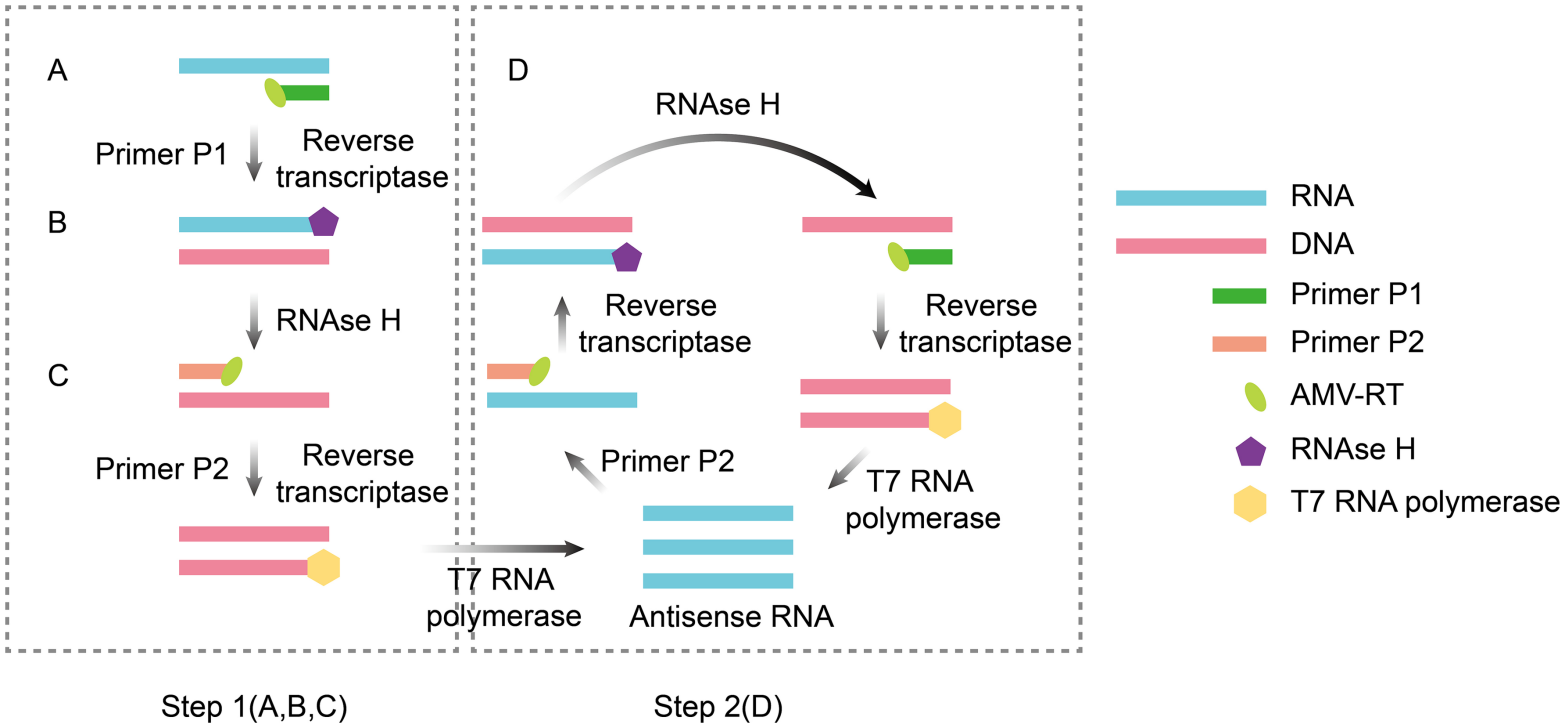
Principle of NASBA amplification. The reaction system contains two primers (F1 and F2) and three enzymes (AMV-RT, RNase H, and T7 RNA polymerase). AMV reverse transcriptase uses RNA as a template to synthesize cDNA (Step 1A), and after hydrolysis of the RNA strand by RNase H (Step 1B), F2 binds to the cDNA to produce double-stranded T7 promoter-containing DNA (Step 1C), which is transcribed by T7 RNA polymerase to produce large amounts of RNA. The new RNA is then used as a template to repeat reverse transcription, hydrolysis, and double-strand synthesis, triggering the T7 transcription loop for self-cycling amplification (Step 2D). The figure was drawn using Adobe Illustrator 2021 software.

NASBA is an isothermal RNA-specific amplification technique that was first proposed by Compton (1991). Its core principle involves achieving exponential amplification of target RNA by copying the natural replication process of viral RNA in host cells. The entire reaction system was maintained at a constant temperature (41°C) and relied on the synergistic action of three enzymes paired with a set of specific primers. Avian myeloblastosis virus (AMV) reverse transcriptase converts RNA templates to cDNA, and RNase H degrades RNA strands in hybridized strands to release single-stranded cDNA. T7 RNA polymerase then transcribes a large amount of single-stranded RNA using promoter-containing double-stranded DNA as a template. In the cyclic phase, the newly synthesized RNA serves as a template for repeating the reverse transcription-degradation-transcription step. Through a noncyclic phase for initial template activation and sustained self-amplification in the cyclic phase, template RNA can be exponentially amplified by a factor of 10^9^ to 10^12^ within 2 h. This method achieves highly efficient target RNA amplification via a continuous, isothermal process (Figure 1). NASBA amplification products can be detected by agarose gel electrophoresis (Jean et al., 2004), microchip electrophoresis (MCE) (Liu et al., 2015), electrochemiluminescence (ECL) (Wacharapluesadee and Hemachudha, 2001) and real-time fluorescence (Aufdembrink et al., 2020).

The emergence of human immunodeficiency virus (HIV) in the 1990s accelerated the development of NASBA, paving the way for faster, more reliable diagnostic methods for HIV tests. The optimization of HIV-1 detection with NASBA Technology was developed by Kievits et al. (1991). Damen et al. quantified hepatitis C virus RNA (HCV-RNA) using nucleic acid sequence-based amplification with quantitative testing (NASBA-QT) and compared the results with those of two commonly used commercial assays (HCV branched DNA assay and HCV MONITOR assay) (Damen et al., 1999). NASBA-QT has demonstrated high sensitivity and reliability in the assay, with a quantitative detection limit of 10^3^ copies/100 μL and a qualitative detection limit of 10^2.3^ copies/100 μL. Compared with the bDNA method, this method has significant advantages (more than a 10-fold increase in sensitivity), and the quantitative results are highly consistent, with comparable sensitivity to that of the HCV MONITOR method, while the latter has systematically lower values (Damen et al., 1999). In addition, Mohammadi-Yeganeh et al. developed a molecular beacon-based multiplex NASBA for the simultaneous detection of HIV-1 and HCV in plasma samples (Mohammadi-Yeganeh et al., 2012). Using the multiplex NASBA technique, Lau et al. successfully detected a wide range of human respiratory viruses, including influenza A, influenza B, respiratory syncytial virus (RSV), and coxsackievirus (CSV) (Lau et al., 2010). Reed et al. developed an innovative label-free colorimetric nucleic acid assay for rapid visual detection of Zika virus (ZIKV) by integrating isothermal nucleic acid amplification with enzymatic signaling mechanisms (Reed et al., 2019). The method demonstrated a detection limit of 10^6^ copies/ml with a clinical sensitivity of 97.64%. The method can be completed within 2 h and selectively differentiates between Dengue and West Nile viruses, which are closely related to the Zika virus (Reed et al., 2019). At the onset of the 2020 COVID-19 pandemic, real-time PCR-based molecular testing became critical for confirming diagnostic results, but its high cost has limited its implementation in resource-poor areas (Wang C. et al., 2020). A real-time NASBA for detecting SARS-CoV-2 was developed by Kia et al. (2023). Rapid amplification of viral RNA at a constant temperature was achieved by designing primers and probes specific to the RdRp and N genes. This method has a detection limit of 200 copies/ml with 97.64% clinical sensitivity, demonstrating performance comparable to that of real-time PCR. Crucially, it eliminates the need for expensive thermal cycling equipment and significantly reduces costs (Kia et al., 2023). Owing to its simple primer design and excellent reproducibility, this method is suitable for rapid screening in resource-limited settings, offering a cost-effective technological alternative to the existing global SARS-CoV-2 assay.

In general, NASBA can avoid DNA interference by directly amplifying RNA targets and is particularly well suited for detecting RNA viruses. However, the NASBA approach has several limitations. First, because the specificity of the reaction depends on thermally unstable enzymes, the reaction temperature should not exceed 42°C. This characteristic also makes the reaction prone to primer dimerization and nonspecific amplification, which increases the likelihood of false positives. Second, the target RNA sequence should be between 120 and 250 nucleotides in length since sequences shorter or longer than this range reduce amplification efficiency (Xue et al., 2022; Liu et al., 2023).

In addition, NASBA is expected to be deeply integrated with molecular beacons, microfluidic technology, etc., to achieve real-time quantitative detection and automated operation. For example, the combination of a G4-ThT fluorescence biosensor and NASBA can detect viral RNA at concentrations as low as 2 copies/μL with real-time detection capability and can be integrated into highly automated systems (such as microfluidic chips) (Guoshuai et al., 2022). The combination of NASBA and the CRISPR system can effectively improve detection sensitivity and specificity. Jung et al. developed and optimized a one-pot NASBA-Cas13a nucleic acid detection method for rapid and sensitive detection of SARS-CoV-2 RNA fragments with a sensitivity of 20–200 aM (Jung et al., 2025). NASBA does not require complex thermal cycling equipment. When combined with a CRISPR portable detection system, it enables the development of low-cost, rapidly deployable field detection tools suitable for remote areas or for responding to sudden epidemics.

## 3 Loop-mediated isothermal amplification

**Figure 2:**
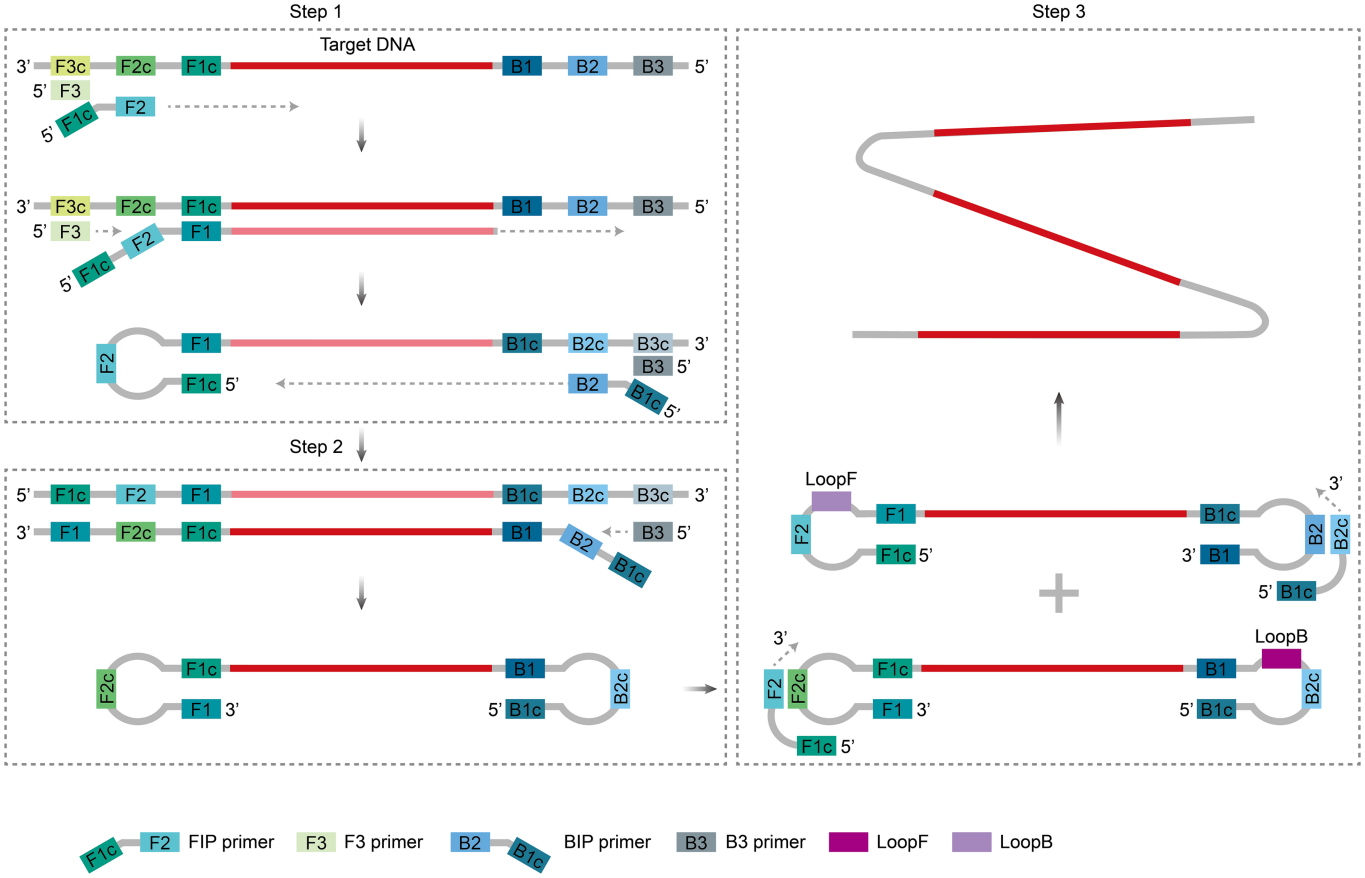
Principle of LAMP amplification. During the initiation phase, the FIP and F3 primers bind sequentially to the target sequence, which is extended by Bst polymerase, displacing the single strand to form a single-stranded loop structure (Step 1); subsequently, the BIP and B3 primers are extended in the reverse direction, generating dumbbell-shaped DNA with loops at both ends that self-fold into a stem–loop template (Step 2). During the cycling phase, the stem–loop template triggers multistep branching amplification through sustained binding, extension, and strand displacement by FIP and BIP primers, forming a polycyclic structure and generating many DNA products containing reverse repeat sequences (Step 3). Two ring primers, LooF and LooB, also bind to the stem–loop structure to accelerate its amplification process. The figure was drawn using Adobe Illustrator 2021 software.

LAMP is a nucleic acid amplification method based on strand substitution developed by Notomi et al. (2000). The core mechanism involves the targeted identification of 6–8 regions of the target gene using 4–6 specific primers (F3/B3, FIP/BIP, LF/LB), followed by efficient amplification by the Bst DNA polymerase (which exhibits strand displacement activity) at a constant temperature of 60–65°C (Figure 2). Loop primers (LF/LB) bind to the stem–loop structure and significantly accelerate the amplification process (Nagamine et al., 2002). Within 30–60 min, exponential amplification of target sequences (up to 10^9^–10^10^-fold) can be achieved. In the presence of reverse transcriptase, the target RNA can also be amplified after being reverse-transcribed into complementary cDNA (Talap et al., 2022). The reaction product comprises numerous alternating, inversely repeated DNA fragments. This result can be detected directly through turbidimetric methods (via magnesium pyrophosphate precipitation) (Mori et al., 2001), fluorescent dye labeling (Soliman and El-Matbouli, 2005), or colorimetric approaches (Tomita et al., 2008), all of which do not require complex instrumentation.

At present, LAMP is widely used in the detection of infectious diseases. Lim et al. (2024) developed a point-of-care testing (POCT) system based on reverse transcription loop-mediated isothermal amplification (RT-LAMP) technology to detect SARS-CoV-2, influenza A and B, and RSV in a single reaction tube. The system combines a nucleic acid-free extraction process with an integrated microfluidic device through multiplex primer design, enabling visual fluorescence interpretation within 30 min. The detection limit was as low as 35–1,000 copies per sample, making it suitable for resource-limited scenarios. However, cost optimization of the device and successful clinical validation are needed to expand its applications. Wang et al. developed a multiplex detection technique combining loop-mediated isothermal amplification and a lateral flow biosensor (LAMP-LFB) for detecting the *Mycobacterium tuberculosis* complex (MTBC) (Wang et al., 2021). The method successfully completed the MTBC assay in less than 1 h, demonstrating a sensitivity of 10 fg. Clinical validation revealed 82% sensitivity and 97.7% specificity in samples, with significantly superior clinical performance compared with bacterial culture (47% sensitivity) and Xpert MTB/RIF (54% sensitivity). This technology has also been employed to detect *Entamoeba histolytica* (pathogenic amoeba), nonpathogenic *Entamoeba* species, pathogenic *Leptospira* spp., *Listeria monocytogenes*, SARS-CoV-2(2 genes), *Enterococcus faecalis* and *Staphylococcus aureus* (Nurul Najian et al., 2016; Foo et al., 2017; Wang et al., 2017; Ledlod et al., 2020; Zhu et al., 2020). Xie et al. integrated LAMP with a self-driven microfluidic chip, achieving automatic pump-less sample loading through the permeability of polydimethylsiloxane (PDMS) material combined with multiple primer design and fluorescence visualization (Xie et al., 2021). This system enables triplex nucleic acid detection of hepatitis B virus (HBV), HCV, and HIV to be completed within 50 min at a constant temperature of 63°C, demonstrating a sensitivity of up to 2 copies/μL. The clinical validation results were completely consistent with the qPCR findings. This method has also been used to detect *Escherichia coli* O157: H7 and *Neisseria meningitidis* (Dou et al., 2014; Guo et al., 2015). Microfluidic chips based on polymethyl methacrylate (PMMA) have been used for the detection of *Escherichia coli* and *Enterococcus* spp., achieving a limit of detection (LOD) of 4 copies per well within 35 min (Fu et al., 2021). This method has also been applied to detect *Vibrio parahaemolyticus* and *Salmonella enterica* (Wu et al., 2021b; Zhu et al., 2023).

With the continuous development of LAMP technology and the integration of various visualization techniques, instant nucleic acid detection has become more accessible and reliable, but the high complexity of LAMP primer design, intricate product structures, cross-interference in multiplex detection and the risk of aerosol contamination remain critical considerations in practical applications (Soroka et al., 2021).

Compared with conventional PCR technology, LAMP generates long DNA strands and cauliflower-like DNA structures, which limits its practicality in molecular biology by producing smeared or multiple bands during gel electrophoresis. In contrast, PCR typically yields single distinct bands. This characteristic makes specific product identification for LAMP more challenging in gel-based analysis. For multiplex detection, traditional multiplex LAMP (mLAMP) strategies, such as restriction enzyme site labeling, often suffer from incomplete enzymatic digestion, resulting in complex band patterns that are difficult to interpret (Crego-Vicente et al., 2024). While molecular barcoding or nanoparticle probe technologies can differentiate targets, they require sequencing or expensive reagents, and they involve time-consuming postprocessing steps and increased contamination risks (Oliveira et al., 2020; Ludwig et al., 2021). Furthermore, the complex product architecture of LAMP prevents target differentiation through melting curve analysis. Probe-based detection methods are also constrained by the inherent strand displacement activity of LAMP (Crego-Vicente et al., 2024). To address these challenges, microfluidic chip technology has demonstrated promise in physically separating primers for different targets into isolated reaction chambers. This spatial segregation effectively minimizes primer competition, streamlines detection workflows, and reduces costs (Liu et al., 2024). Such microfluidic platforms may overcome current limitations to enable efficient, cost-effective, and scalable mLAMP solutions. Additionally, the complexity of primer design introduces uncertainties into detection outcomes. Although Primer Explorer V5 remains the most widely used online tool for LAMP primer design, it occasionally fails to identify optimal targets automatically, necessitating labor-intensive manual optimization. The substantial length of key primers (FIP/BIP: 30–40 bases) and the requirement for multiple primer sets dramatically increase the risk of self-hybridization and nonspecific amplification, potentially causing false-positive results. Avoiding this risk often demands iterative experimental validation to optimize primer combinations (Soroka et al., 2021).

## 4 Recombinase polymerase amplification

**Figure 3:**
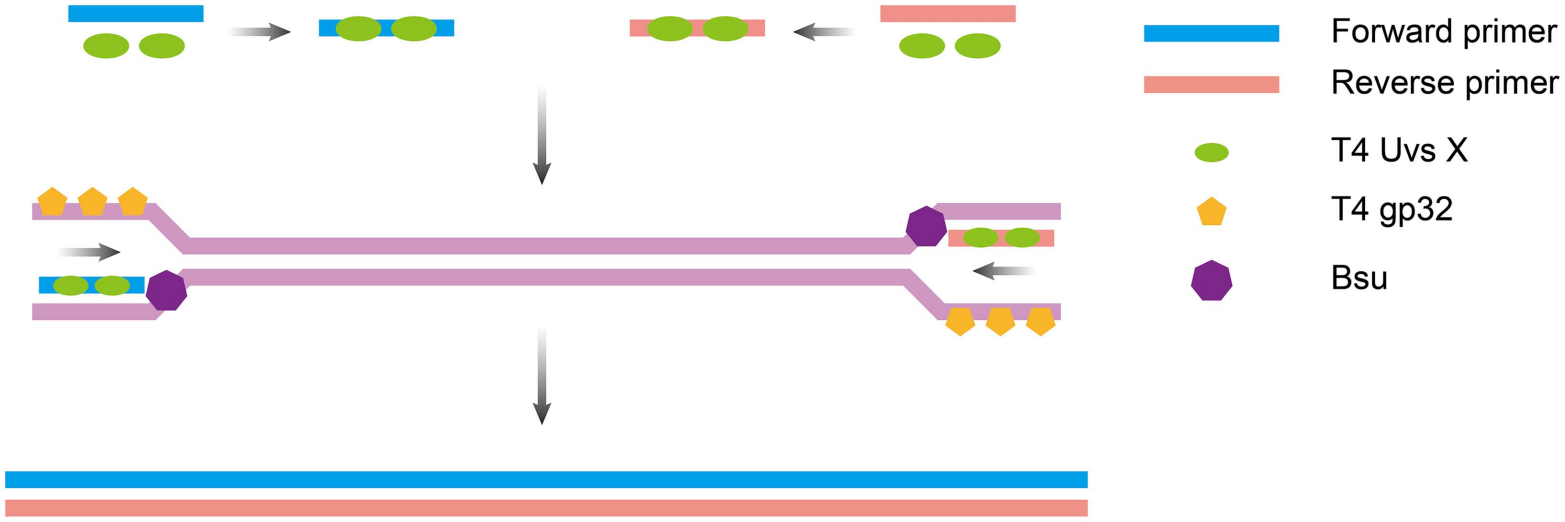
Principle of RPA amplification. Recombinase-primer complexes anneal to double-stranded DNA, inducing localized strand separation to generate single-stranded DNA regions; single-strand binding proteins stabilize the template, and strand-displacing polymerase extends the primer and displaces the complementary strand, creating a new double strand. The displaced strand serves as a template for subsequent amplification cycles, driving recombinase complex binding and strand synthesis. The figure was drawn using Adobe Illustrator 2021 software.

RPA, an isothermal nucleic acid amplification technology first developed in 2006 by a team led by the British scientist Piepenburg et al. (2006), mimics the mechanism of viral DNA replication. It employs recombinant enzymes (e.g., the T4 UvsX protein) to guide primers toward double-stranded DNA binding with single-stranded binding proteins (e.g., T4 gp32) to stabilize the template, and strand-displacing polymerases (e.g., Bsu polymerase). The system enables rapid exponential amplification of target sequences at a constant temperature of 37–42°C, requiring only 20–30 min to amplify trace amounts of nucleic acids (as low as 1–10 copies) to detectable levels (Figure 3). The entire process requires no thermal cycling equipment and relies on the synergy of the enzyme system to achieve efficient and rapid nucleic acid replication. The amplification products generated from RPA can be detected via endpoint (post- amplification) or real-time (during amplification) assays, with RPA coupled with a lateral flow strip (RPA-LFS) and real-time RPA being two of the most widely used methods.

RPA-LFS has been widely adopted for infectious disease testing because it allows for rapid visual interpretation of results with the naked eye. Ji et al. developed a rapid RPA-LFS-based assay for *Staphylococcus haemolyticus* that achieved nucleic acid amplification in 8 min and 1 min of visual interpretation. The assay demonstrated a sensitivity of 0.147 CFU/μL and showed 100 and 98.73% consistency with qPCR and conventional culture, respectively, in validation with clinical samples (Ji et al., 2023). RPA-LFS is also applicable for detecting *Streptococcus pneumoniae* with high sensitivity (3.32 CFU/μL) and specificity within 15 min, and the clinical sample validation results were highly concordant with those of PCR (98.18% concordance rate). In addition, RPA-LFS shows no cross-reactivity with other hepatitis viruses, with the lowest detection limits of 10 copies/ml for HBV and 100 copies/ml for hepatitis E viruses (HEVs) (Zhang et al., 2021; Li M. et al., 2023). In real-time RPA, fluorescent probes are combined to enable real-time monitoring of RPA amplification of target sequences. Zhang et al. developed a real-time RPA assay for *Yersinia enterocolitica* detection in intestinal samples, achieving detection within 20 min with a sensitivity of 10^4^ ng/μL (Zhang et al., 2023). Ying et al. applied real-time RPA for subtyping human papillomavirus (HPV) 16 and 18, achieving 1,000 copies/μL sensitivity (Ying et al., 2023).

RPA technology offers several unique advantages during the detection of infectious diseases, including simple primer design, streamlined amplification protocols, rapid reaction kinetics, and high sensitivity and specificity. It supports the detection of both DNA and RNA targets (when combined with reverse transcriptase) (Lobato and O'Sullivan, 2018) and is compatible with multiple detection methods, such as fluorescent probes, lateral flow dipsticks, and integration with CRISPR-based systems (Kellner et al., 2019). However, as a relatively new method with a short development history, RPA also has certain limitations. For example, its amplification fragment length is typically restricted to 100–500 bp, which hinders long-sequence analysis. Primer design requires rigorous structural optimization to avoid nonspecific amplification. Low and isothermal amplification of the RPA can easily lead to false positives and must be combined with other technologies to improve specificity. Zhang et al. developed a method by combining the CRISPR/Cas12a/13a systems, leveraging the cleavage specificity of Cas12a and Cas13a to detect HPV16 and HPV18 simultaneously with high specificity and sensitivity (10 copies/μL) (Zhang et al., 2024). Additionally, the current limitations in multichannel parallel processing capability impede its efficient application in large-scale screening scenarios (Lobato and O'Sullivan, 2018; Wu et al., 2024). Despite these limitations, further exploration and enhancement of its strengths could position RPA as a mainstream nucleic acid amplification technology in the future.

## 5 Recombinase-aided amplification

**Figure 4:**
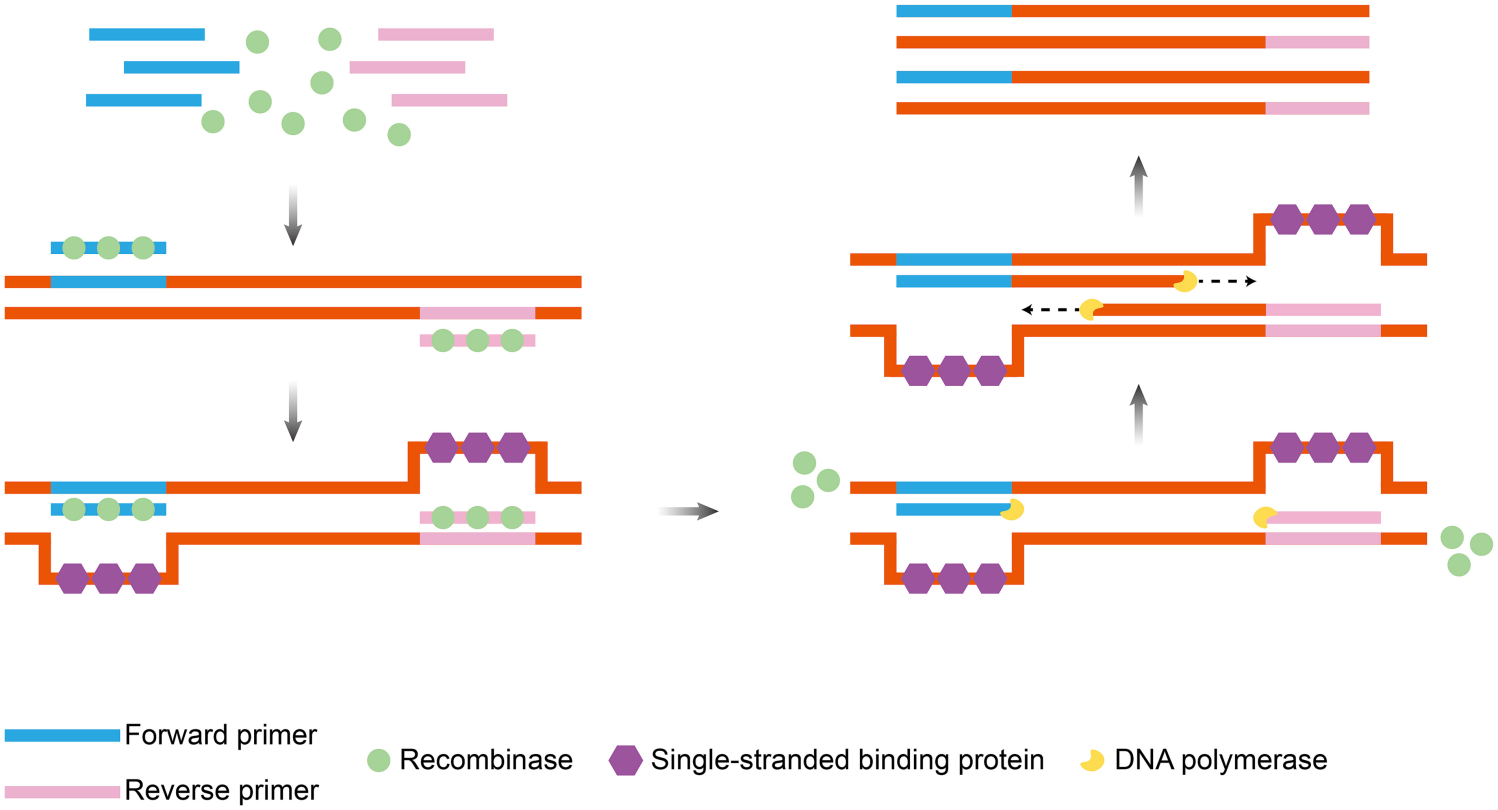
Principle of RAA amplification. Under isothermal conditions, recombinase, single-strand binding protein and DNA polymerase operate synergistically: recombinase targets and binds to specific DNA sequences, induces strand unwinding, single-strand binding protein stabilizes the single-stranded template, and DNA polymerase catalyzes strand extension to drive exponential amplification of target DNA. The figure was drawn using Adobe Illustrator 2021 software.

RAA technology is an isothermal nucleic acid amplification technology proposed and optimized by a Chinese research team in 2010 (Shen et al., 2019). RAA employs recombinant enzymes to recognize target sequences and unwind double-stranded DNA (typically derived from fungi or bacteria, such as bacterial RecA or engineered enzymes). This process enables single-stranded binding proteins to protect the template and facilitates DNA polymerase-mediated strand exchange synthesis of the new strand. This synthesis occurs in conjunction with bispecific primers and fluorescent probes for real-time monitoring (Nie et al., 2022). The entire amplification process shares key characteristics with RPA, including operation at a constant temperature (37–42°C) and rapid completion (within approximately 30 min) (Figure 4). The final product, a mixture of double-stranded DNA and single-stranded DNA, can be directly detected using methods such as fluorescent probes, lateral flow strips, or electrophoresis (Wu K. et al., 2021; Lin et al., 2022; Wang K. et al., 2022).

RAA represents an advanced isothermal amplification technique. With technological advancements in RAA, the development of RAA-derived variants has enabled more convenient and rapid detection approaches for infectious diseases. For example, Bai et al. developed a real-time fluorescent RAA-based assay for HBV that can be performed in a single tube at 39°C for 40 min, with a minimum detection limit of 100 IU/mL, a sensitivity of 97.18%, and a specificity of 100% for clinical samples (Bai et al., 2020). To detect methicillin-resistant *Staphylococcus aureus* (MRSA), real-time RAA fluorescence technology has a detection limit of 10 copies/μL, with 97.01% consistency with traditional qPCR results, and high specificity without cross-reactivity with other clinically relevant bacteria (Ding et al., 2022). Li et al. developed a reverse transcription-recombinase-assisted amplification (RT-RAA)-based POCT assay for SARS-CoV-2 nucleic acids in combination with a lateral flow strip (LFS) (Zheng et al., 2021). Through specific targeting of the viral nucleocapsid (N) gene, the assay was performed at 39°C for 30 min, with a limit of detection of 1 copy/μL. Method validation revealed no cross-reactivity with human coronavirus, influenza A/B virus, RSV, or HBV. The clinical evaluation of 100 samples (13 positive, 87 negative) demonstrated 100% sensitivity and specificity compared with RT-qPCR, with advantages such as rapidity, high precision, and room-temperature operation, highlighting its suitability for POCT applications. It has been validated for detecting avian influenza virus subtype H5 (Li Y. et al., 2023), senecavirus A (Wang W. et al., 2022) and HCV (Wang H. et al., 2022). Integration with the CRISPR/Cas system enhances nucleic acid detection specificity for low-copy-number targets. For example, Qian et al. combined the CRISPR/Cas12a system with RT-RAA isothermal amplification to achieve highly sensitive (0.1 copies/μL), rapid (30–40 min) detection of norovirus subtypes GII.4 and GII.17 without complex equipment, demonstrating over 95% clinical concordance with clinical samples and showing no cross-reactivity with related viruses (Qian et al., 2022).

The RAA assay, a novel isothermal amplification technique, has been widely used for detecting various pathogens. Unlike RPA assays, RAA technology has been optimized in the formulation to adapt to China’s pathogen detection. The RAA employs three core enzymes: recombinase UvsX, DNA polymerase, and single-stranded DNA-binding protein (Xue et al., 2020). A key advantage of RAA technology lies in its ability to perform amplification under optimized conditions at 37°C or even room temperature, enabling the acquisition of target amplification products within 30 min. This process achieves exponential amplification of target DNA without requiring auxiliary heating equipment, making it particularly suitable for on-site pathogen detection. Furthermore, the RAA reaction system mandates strict complementarity between primers and templates, with primer lengths restricted to 30–35 nucleotides. This stringent requirement ensures both the precision and specificity of the RAA method, distinguishing it from other amplification approaches.

However, owing to the very high sensitivity of RAA, which can easily cause false-positive results, cross-contamination should be avoided throughout the process. This prevention requires vigilant control throughout sample collection, reagent preparation, amplification reactions, and result analysis. Key recommendations include maintaining separate laboratory zones (e.g., sample handling, reagent preparation, and amplification areas) with proper ventilation; using disposable, nuclease-free consumables and filtered pipette tips; practicing aseptic techniques (e.g., gentle pipetting, frequent glove changes); aliquoting reagents to avoid repeated thawing; incorporating negative and positive controls; optimizing primer/probe specificity; and implementing regular training for personnel. Stringent adherence to these protocols, coupled with standardized operating procedures, can effectively mitigate contamination risks and ensure accurate, reliable RAA results. In addition, nonspecific products may be generated during the reaction because of the short length of the target genes, and primer dimer formation may be caused by excess primers and specific interactions between primer molecule (Kellner et al., 2019; Wang et al., 2019). As with other IATs, the practical application of RAA technology also faces the same challenges. First, achieving on-site sample preprocessing (e.g., nucleic acid extraction) in environments requiring immediate sample processing remains difficult; however, this step is critical for obtaining high-quality nucleic acid templates essential for amplification. Second, achieving highly sensitive and specific multitarget isothermal amplification detection under single closed-tube conditions presents a significant technical hurdle. The ability to detect multiple targets in a single reaction simultaneously is vital for efficient and comprehensive pathogen identification. To broaden the applicability of RAA and fully unlock its potential, current research and development efforts are focused on addressing these issues to increase the practicality and effectiveness of RAA in real-world settings.

## 6 Isothermal nucleic acid amplification combined with the CRISPR/Cas system

**Figure 5:**
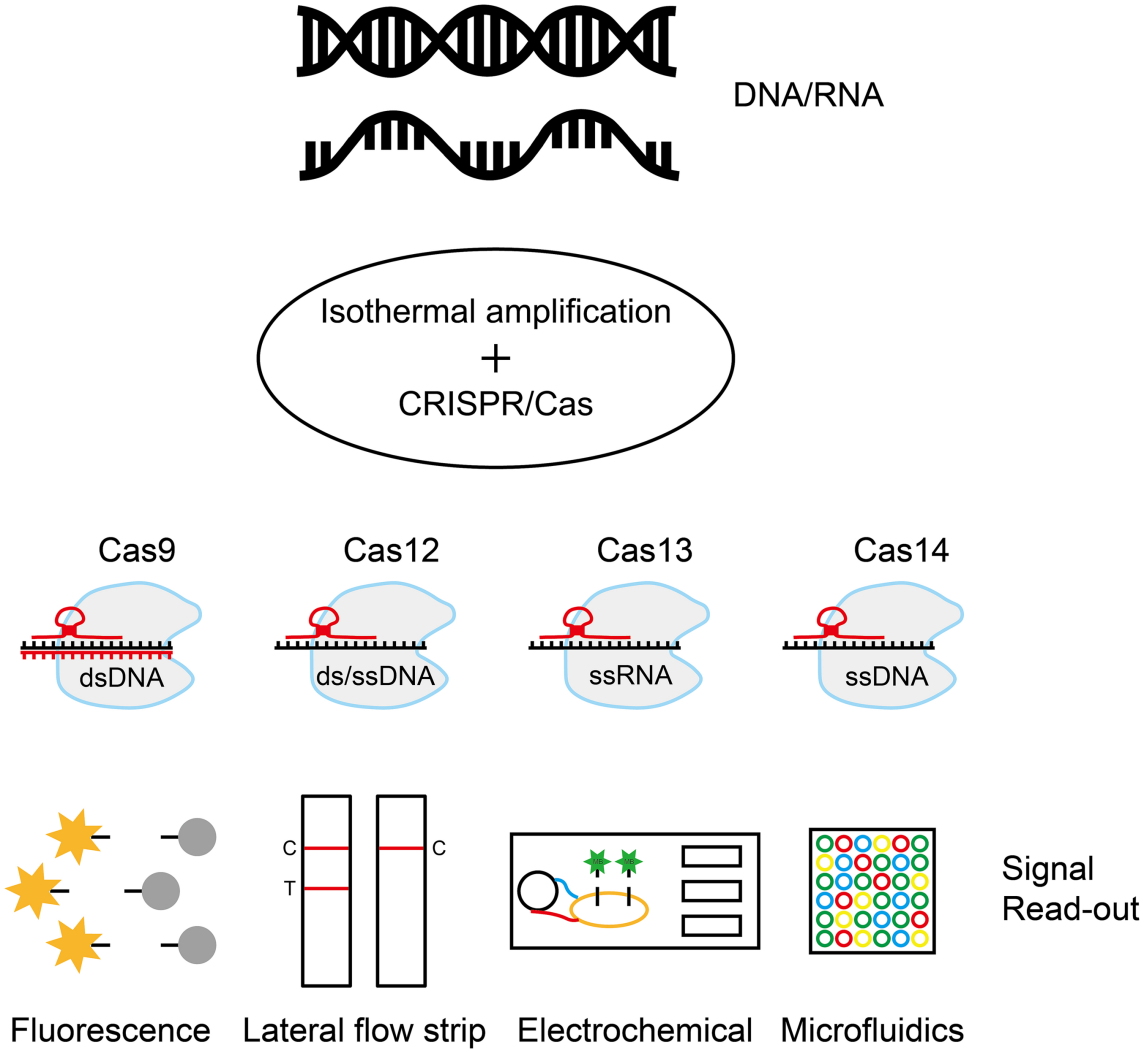
Isothermal amplification combined with the CRISPR/Cas system. Through rapid isothermal amplification of DNA/RNA, the CRISPR/Cas system performs targeted cleavage of the target sequence and signal recognition using downstream techniques such as fluorescent labeling, lateral chromatography test strips, or electrochemical signaling. The figure was drawn with Adobe Illustrator 2021 software.

Isothermal nucleic acid amplification combined with the CRISPR/Cas system is a molecular diagnostic tool adapted from the bacterial immune system. The clustered regularly interspaced short palindromic repeats sequence (CRISPR) system, which evolved as a bacterial defense mechanism against viral invasion, consists of the enzyme Cas nuclease and a guide RNA (gRNA). The gRNA recognizes a specific DNA or RNA sequence, and the Cas enzyme precisely cuts the target (Makarova and Koonin, 2015). The research team combined the system’s targeted cleavage capability with isothermal amplification technology. Once the Cas protein recognizes the target sequence in a sample, it triggers its cleavage activity, which in turn enables visual detection using downstream techniques such as fluorescent labeling, lateral flow test strips, or electrochemical signaling (Figure 5) (Gootenberg et al., 2017; Kaminski et al., 2021).

### 6.1 Combined CRISPR/Cas9 and isothermal amplification of nucleic acids

Pardee et al. combined NASBA with the CRISPR/Cas9 system to develop a molecular diagnostic assay called NASBA-CRISPR cut (NASBACC), which discriminates ZIKV genotypes (e.g., American vs. African strains) with single-base resolution (Pardee et al., 2016). In this assay, researchers used NASBA to amplify viral RNA and incorporated trigger sequences that activate a toehold switch sensor into the amplification products. gRNAs were designed to target single-nucleotide polymorphism (SNP) sites specific to the American strain, which contains NGG protospacer adjacent motif (PAM) sequences. Cas9 recognized the PAM site of the American strain and cleaved the amplified DNA, resulting in the truncation of the RNA product, a lack of a trigger sequence to activate the sensor, and the maintenance of the original color (yellow). In the African strain, the absence of the PAM sequence prevented Cas9-mediated cleavage, leaving the RNA product intact and harboring a trigger sequence that was bound to the toehold switch, activating *LacZ* reporter gene expression and inducing a color change from yellow to purple (Pardee et al., 2016). This method was used to identify Zika virus in the Americas and Zika virus in Africa.

The CRISPR/Cas9-mediated lateral flow nucleic acid assay (CASLFA) integrates the CRISPR/Cas9 system, lateral flow assay (LFA), and RPA/PCR-based DNA/RNA detection methods (Wang et al., 2020b). It uses a Cas9/sgRNA complex to bind to target double-stranded DNA and releases nontarget single-stranded regions. These regions hybridize with a gold nanoparticle (AuNP) probe, generating a visual signal on a lateral flow strip. CASLFA can detect *Listeria monocytogenes*, transgenic rice (35S promoter), and African swine fever virus (ASFV) with limits as low as hundreds of genome copies, and when combined with RPA, sample-to-result analysis can be completed in less than 40 min. In validation, CASLFA accurately identified 27 ASFV-positive samples among 110 swine serum suspects, showing 100% concordance with real-time PCR results in terms of sensitivity and specificity (Wang et al., 2020b). Additionally, the CRISPR/Cas9-mediated triple-line lateral flow assay (TL-LFA), combined with multiplex reverse transcription RPA (RT-RPA), enables rapid simultaneous detection of the SARS-CoV-2 E gene and Orf1ab gene on a single test strip. This method achieves a sensitivity of 100 RNA copies per 25 μL reaction system. Clinical validation using 64 nasopharyngeal samples demonstrated 100% negative predictive agreement and 97.14% positive predictive agreement (Xiong et al., 2021). This approach offers an accurate and convenient diagnostic solution for detecting COVID-19 in resource-limited settings.

### 6.2 Combined CRISPR/Cas12 and isothermal amplification of nucleic acids

The DNA endonuclease-targeted CRISPR trans reporter (DETECTR), developed by Chen et al. in 2018, is a molecular diagnostic technology that integrates isothermal RPA with Cas12a enzymatic activity (Chen et al., 2018). The target sequence is amplified by RPA, and the gRNA directs the Cas12a protein to recognize and bind to the target sequence. Upon target engagement, Cas12a cleaves the double-stranded DNA (cis cleavage) and simultaneously activates trans cleavage activity, releasing fluorescent signals through nonspecific cleavage of the free single-stranded DNA (ssDNA) probe. Xu et al. developed a rapid detection method for *Bacillus anthracis* based on DETECTR, achieving fast (<40 min), highly sensitive (nearly two-copy level), and specific detection of the pathogen’s nucleic acid (Xu et al., 2023). This method also enabled the discriminatory detection of HPV16/18 (Chen et al., 2018), Japanese encephalitis virus genotypes I, III, and V (Kwak et al., 2023) and African swine fever virus (Wang et al., 2020a), demonstrating its versatility in differentiating infections caused by these pathogens. OR-DETECTR is an assay based on RT-RPA and DETECTR technology. Researchers have optimized the reaction components such that the assay can be performed in a single test tube, requires no special equipment, and can be completed in less than 1 h. It has been used to detect the H1N1 and SARS-CoV-2 viruses (Sun et al., 2021).

Lee et al. established a rapid, sensitive and visual detection method for the *Escherichia coli* O157: H7 gene using the LAMP-CRISPR/Cas12a system (Lee and Oh, 2022). The target gene sequence is amplified by LAMP. Cas12a binds to the amplified sequence and then cleaves the fluorescently labeled ssDNA probe, releasing a fluorescent signal. This approach has also been reported for detecting *Salmonella* spp., *Neisseria meningitidis* and *Pseudomonas aeruginosa* (Mukama et al., 2020; Wu et al., 2021a).

### 6.3 Combined CRISPR/Cas13 and isothermal amplification of nucleic acids

SHERLOCK (Specific High-sensitivity Enzymatic Reporter UnLOCKing) is a molecular diagnostic method based on the CRISPR-LWA-Cas13a system developed by Feng Zhang’s team in 2017 (Gootenberg et al., 2017). This method uses the targeted RNA recognition ability and cleavage activity of the Cas13a protein to achieve highly sensitive and specific detection of DNA or RNA target sequences. These sequences are amplified using RPA (for DNA) or RT-RPA (for RNA), with DNA amplicons transcribed to ssRNA by T7 RNA polymerase. gRNA guides the LwaCas13a protein to recognize target sequences, activating nonspecific cleavage of fluorescent ssRNA reporter probes to generate detectable signals. SHERLOCK technology has been employed in the detection of the Zika and Dengue viruses, demonstrating high sensitivity and specificity (Gootenberg et al., 2017). Allan-Blitz et al. developed a dual-molecule diagnostic system based on the CRISPR/Cas13a-powered SHERLOCK platform. This system enables pathogen detection by targeting the porA gene (with a sensitivity of 14 strains of *Neisseria gonorrhoeae* and no cross-reactivity with 3 non-gonococcal *Neisseria* strains) while simultaneously predicting ciprofloxacin resistance through the identification of mutations in the gyrase A (gyrA) gene (91/91 mutation sites validated using 20 resistant strains and 3 susceptible strains, with DNA sequencing confirming 100% concordance) (Allan-Blitz et al., 2023).

However, SHERLOCK technology still faces limitations, including the lack of quantitative capability and reliance on fluorescence detection equipment for readout. Therefore, the team further optimized this approach in 2018 by developing multiple detection capabilities based on the first generation, with higher sensitivity and more flexible application scenarios (Gootenberg et al., 2018). SHERLOCKv2 allows for the simultaneous detection of four different target nucleic acids (DNA or RNA) by employing four different Cas proteins (LwaCas13a, PsmCas13b, CcaCas13b and AsCas12a). Each Cas protein is guided by a specific crRNA. Upon target recognition and activation, each Cas protein cleaves various types of reporter probes, such as fluorescently labeled RNA or DNA, enabling parallel multitarget detection. Furthermore, by incorporating the Csm6 protein (a CRISPR-associated nuclease), the system achieves signal amplification. When Cas13 cleaves its target RNA, it releases fragments that activate Csm6. Activated Csm6 then cleaves additional reporter probes, amplifying the signal through a cascade reaction and increasing the sensitivity to 2 aM. SHERLOCKv2 combines fluorescence detection with lateral flow strips (colorimetric readouts), offering quantification, visual interpretability, and flexible multiformat reporting, thereby expanding its utility in nucleic acid diagnostics (Gootenberg et al., 2018).

To enable large-scale multiplexed nucleic acid detection, Ackerman et al. developed a high-throughput platform using CARMEN-Cas13 (Ackerman et al., 2020). The core mechanism enables simultaneous differentiation of 169 human-associated viruses by PCR or RPA amplification of target sequences followed by the use of microfluidic arrays with the trans-cleaving activity of Cas13. Simultaneously, comprehensive subtyping of influenza A strains and multiple identification of dozens of HIV drug-resistant mutations have been achieved (Ackerman et al., 2020).

### 6.4 Combined CRISPR/Cas14 and isothermal amplification of nucleic acids

Cas14-DETECTR, a Cas14-based DNA detection platform developed by Aquino-Jarquin et al. in 2019, integrates Cas14’s highly specific ssDNA recognition with trans-cleavage activity for sensitive and specific nucleic acid detection (Harrington et al., 2018; Aquino-Jarquin, 2019). The workflow involves RPA amplification of target sequences, crRNA-guided specific binding of the Cas14 protein to ssDNA targets, and activation-driven nonspecific cleavage of the fluorescent ssDNA reporter probes. The Cas14-DETECTR method has been shown to detect the human E3 ubiquitin protein ligase (HERC2) gene accurately (Harrington et al., 2018). The Cas14 protein exhibits high-fidelity target sequence recognition, making Cas14-DETECTR a high-fidelity detection tool for identifying medically important pathogens and SNPs (Aquino-Jarquin, 2019). Chen et al. developed a naked-eye colorimetric assay using multiplexed isothermal amplification with CRISPR/Cas14a for aflatoxin B1 (AFB1), employing magnetic/gold nanocomposite probes (MAPs) (Chen et al., 2023). This technology uses an aptamer competition mechanism to capture the triggered toxin, triggers a DNA signal amplification reaction and produces a visual color change by CRISPR/Cas14a-specific cleavage of MAPs, with a detection limit of 31.90 pg./mL. It demonstrates excellent specificity and accuracy in real-world samples. To address the challenges of *Helicobacter pylori* antibiotic resistance detection, Lai et al. developed the Cas14VIDet visual detection platform, which integrates ultrarapid PCR with the CRISPR/Cas14 system in a single-tube reaction format (Lai et al., 2025). This innovative approach overcomes the limitations of conventional methods by enabling the precise identification of SNPs without requiring a PAM sequence. The platform achieves exceptional sensitivity at 100 CFU/mL (single-colony level) and allows for visual interpretation of resistance genes within 10 min. When validated with 50 clinical samples, it demonstrated 100% sensitivity, specificity, and accuracy in detecting levofloxacin resistance genes, showing complete consistency with the Sanger sequencing results. This breakthrough provides an efficient solution for guiding precise antibiotic selection in *H. pylori* infection management.

In summary, the IAT-CRISPR/Cas system leverages its highly specific nucleic acid recognition ability by combining isothermal amplification and signal conversion mechanisms to achieve efficient detection of various pathogens. This approach offers high sensitivity, low cost, and simple operation, making it suitable for POCT applications (Shao et al., 2019; Mao et al., 2023). However, CRISPR/Cas systems still face several challenges in nucleic acid detection. First, owing to the off-target effects of the CRISPR/Cas system, gRNAs can recognize and induce cleavage at nontarget DNA or RNA sequences, leading to false-positive test results. To mitigate this problem, strategies include optimizing the Cas protein and CRISPR/Cas base sequences to obtain more stable mutants and adjusting the gRNA sequence length, mismatch tolerance, GC content, and reaction conditions (Hajiahmadi et al., 2019). Second, the recognition of target sequences by Cas effector proteins is limited by specific PAM sequences, restricting their widespread application. Solutions involve introducing PAM sequences into PCR and LAMP amplification products, using PAM-containing primers, or developing CRISPR/Cas systems with broader PAM recognition (Harrington et al., 2018; Li et al., 2018). Lastly, the activity of CRISPR/Cas systems directly affects detection sensitivity, requiring continuous optimization of Cas proteins and corresponding gRNAs or crRNAs to increase system activity (Hajiahmadi et al., 2019; Arizti-Sanz et al., 2020).

In addition, when nucleic acid amplification is coupled with CRISPR/Cas detection systems, CRISPR/Cas-based diagnostics face challenges in commercialization, contamination risks, and insufficient sensitivity. The key issue lies in effectively amplifying nucleic acid signals to levels detectable by Cas systems. To address this need, CRISPR/Cas12-based diagnostic technologies (e.g., SHERLOCK) can be optimized into independent steps of nucleic acid amplification and CRISPR/Cas detection to increase sensitivity, but this optimization increases procedural complexity and cross-contamination risks. Alternatively, single-step nucleic acid detection strategies exhibit lower sensitivity, whereas two-step approaches suffer from operational complexity and commercialization hurdles. Future advancements aim to integrate CRISPR/Cas systems into single-reaction formats for improved efficiency. For example, Hu et al. developed a light-controlled single-tube RPA-CRISPR/Cas12a DNA detection system that demonstrated promising sensitivity (Hu et al., 2022). One pot of SHINE (Streamlined Highlighting of Infections to Navigate Epidemics) enables visualized fluorescence readouts, reducing contamination rates and facilitating interpretation through a smartphone application, but it still lacks robust clinical evidence, comprehensive functionality, and scalability for widespread clinical applications (Arizti-Sanz et al., 2020). Future advancements should focus on device miniaturization, optimizing multiplex detection technologies, expanding clinical applications across diverse scenarios, and establishing standardized protocols (Gootenberg et al., 2018; Kaminski et al., 2021).

## 7 Conclusion

IAT is a method for rapid amplification of nucleic acid fragments at a constant temperature, which enables efficient and sensitive amplification of DNA/RNA through a specific enzyme-catalyzed reaction without thermal cycling. Based on their operational simplicity and rapid reaction kinetics, IATs are suitable for immediate detection and resource-limited environments, including rapid on-site diagnostic and pathogen detection. The comparative technical specifications of different IATs (Table 1) and conventional PCRs (Table 2) visually demonstrate the advantages of the IAT in terms of convenience, efficiency and sensitivity for pathogen detection. Therefore, IAT is considered a highly efficient approach for rapid POCT. In addition, combining IATs with the CRISPR/Cas system enables rapid and sensitive molecular detection by integrating the precise recognition capability of the CRISPR gene editing tool with the high efficiency of isothermal nucleic acid amplification technology. Table 3 shows the application of different Cas systems combined with IATs for the detection of pathogens and related technical indicators (Table 3).

**Table 1:** Key metrics comparison of isothermal amplification technologies.

Technology	Time (min)	Sensitivity	Sample types	Detection method	Cost (vs. PCR)	Advantages and disadvantages	Detected pathogens	References
NASBA	60–90	10^2^–10^3^ copies/μL	Plasma, respiratory samples	Electrophoresis, fluorescence, colorimetry	Moderate	**Advantages**: Direct RNA amplification; Compatible with CRISPR for SNP typing **Disadvantages**: Temperature-sensitive (≤42°C); Prone to primer dimerization	HIV-1, HCV, Zika virus, Influenza A/B, SARS-CoV-2	van Gemen et al. (1993), Damen et al. (1999), Deiman et al. (2002), Reed et al. (2019), Wang C. et al. (2020), Ngoc and Lee (2024)
LAMP	30–60	1–10 copies/μL	Blood, sputum, environmental water	Turbidity, fluorescence, LFS	Low	**Advantages**: High specificity (multi-primer design); Visual readout **Disadvantages**: Complex primer design; Aerosol contamination risk	*Mycobacterium tuberculosis*, SARS-CoV-2, HBV, HCV, HIV, *Listeria monocytogene*, *Neisseria meningitidis*	Guo et al. (2015), Ledlod et al. (2020), Soroka et al. (2021), Wang et al. (2021), Xie et al. (2021), Lim et al. (2024), Ngoc and Lee (2024)
RPA	20–40	1–10 copies/μL	Whole blood, stool, food	LFS, fluorescence	Moderate	**Advantages**: Rapid; Room-temperature operation **Disadvantages**: Limited amplicon length (100–500 bp); Higher cost of reagents	*Staphylococcus haemolyticus*, *Streptococcus pneumoniae*, *Yersinia enterocolitica*, HBV, HEV, HPV16/18	Lobato and O'Sullivan (2018), Zhang et al. (2021), Tan et al. (2022), Ji et al. (2023), Li M. et al. (2023), Ying et al. (2023), Zhang et al. (2023)
RAA	30–40	1–10 copies/μL	Serum, swab, tissue	Fluorescence, LFS	Low	**Advantages**: Localized adaptability; High sensitivity **Disadvantages**: Contamination risks; Primer dimer issues	Methicillin resistant *Staphylococcus Aureus* (MRSA), SARS-CoV-2, Avian influenza H5, Senecavirus A, HCV	Bai et al. (2020), Wang et al. (2020b), Zheng et al. (2021), Ding et al. (2022), Wang K. et al. (2022), Wang W. et al. (2022), Li X. et al. (2023), Li Y. et al. (2023)
CRISPR/based	40–120	0.1–10 copies/μL	Complex matrices (e.g., soil, blood)	LFS, Electrochemistry	Moderate	**Advantages**: Single-base resolution; Multiplex detection **Disadvantages**: Off-target effects; Commercialization challenges	Zika, Dengue, SARS-CoV-2, ASFV, Norovirus, *Listeria monocytogenes*, *Escherichia coli* O157: H7, *Salmonella*	Pardee et al. (2016), van Dongen et al. (2020), Wang et al. (2020b), Sun et al. (2021), Lee and Oh (2022), Zhou et al. (2024)

**Table 2:** Isothermal amplification technologies vs. traditional PCR.

Metric	Isothermal amplification (e.g., LAMP/RPA)	Traditional PCR
Equipment	Constant-temperature block/water bath, portable devices	Thermal cycler, lab equipment
Processing time	20–90 min	2–3 h (including cycling)
Sensitivity	Comparable (higher for CRISPR systems)	High (1–10 copies/μL)
Multiplexing	Supported in some systems (e.g., SHERLOCKv2)	Requires complex primer design
Cost	Low (reagents + simple devices)	High (equipment + consumables)
Application	Field testing, resource-limited settings	Standardized laboratories
Technical skill	Low (minimal training required)	High (requires expertise)
Advantage	Short time, suitable for POCT Simple equipment and low cost, suitable for resource-constrained scenarios Highly sensitive and visualized assays (e.g., turbidity/fluorescence)	Mature technology with high amplification efficiency Long fragment amplification capability, suitable for sequencing and cloning High throughput compatibility (e.g., qPCR, NGS)
Disadvantage	Short fragment amplification limitation, not applicable to long gene analysis Open system is easy to be contaminated, need to be strictly operated Easy to generate non-specific fragments Weak high-throughput detection capability	Relies on sophisticated equipment and is complicated to operate Long reaction time, not suitable for immediate detection Limited accessibility at the grassroots level
References	Zhao et al. (2015), Boonbanjong et al. (2022), Srivastava and Prasad (2023)	Smith and Osborn (2009), Schrader et al. (2012), Munawar (2022)

**Table 3:** CRISPR/Cas systems combined with isothermal amplification technologies.

Technology name	Cas type	IAT	Sensitivity	Time	Signal readout	Advantages	Limitations	Detected pathogens	References
NASBACC	Cas9	NASBA	Single-base resolution	~2 h	Colorimetric (yellow → purple)	High specificity for SNP differentiation, visual results	Complex sensor design, temperature sensitivity optimization required	Zika virus	Pardee et al. (2016)
CASLFA	Cas9	RPA/PCR	Hundreds of genomic copies	40 min	Lateral flow strip (naked eye)	Rapid, equipment-free, high sensitivity	Multi-step workflow, primer design dependency	*Listeria monocytogenes*, ASFV, SARS-CoV-2	Wang et al. (2020b), Xiong et al. (2021)
LAMP-CRISPR	Cas12a	LAMP	1.22 CFU/mL	1 h	Fluorescence or colorimetric	Rapid visualization, field-deployable	Aerosol contamination risks, primer design complexity	*Escherichia coli* O157: H7, *Salmonella* spp.	Mukama et al. (2020), Lee and Oh (2022)
DETECTR	Cas12a	RPA	116 copies/μL	1 h	Fluorescence or lateral flow	Single-tube reaction, high sensitivity, multiplex compatibility	Limited amplicon length (<500 bp), contamination risks	HPV16/18, ASFV, *Bacillus anthracis*	Chen et al. (2018), Wang et al. (2020a), Xu et al. (2023)
OR-DETECTR	Cas12a	RT-RPA	2.5 copies/μL	1 h	Fluorescence or lateral flow	Single-tube workflow, rapid screening	Lower sensitivity than PCR, reverse transcription required	SARS-CoV-2, H1N1 influenza virus	Sun et al. (2021)
SHERLOCK	Cas13a	RPA/RT-RPA	2 aM	1–2 h	Fluorescence or colorimetric	Ultra-high sensitivity, quantitative capability	Requires fluorescence reader, multiplex optimization challenges	Zika virus, dengue virus, SARS-CoV-2	Gootenberg et al. (2017), Joung et al. (2020)
SHERLOCKv2	Cas13a/b, Cas12a	RPA/RT-RPA	0.1–2 aM	1–2 h	Fluorescence, colorimetric	4-plex detection, signal amplification (Csm6 cascade), portability	High commercialization costs, device miniaturization needed	Zika virus, Dengue virus, *Pseudomonas aeruginosa*, *Staphylococcus aureus*	Gootenberg et al. (2018)
CARMEN-Cas13	Cas13	RPA/PCR	Picogram-level	~4 h	Microfluidic array fluorescence	High-throughput, large-scale multiplex detection	Complex instrumentation, high cost	SARS-CoV-2, Influenza A subtypes, HIV, Hepatitis B/C viruses	Ackerman et al. (2020)
Cas14-DETECTR	Cas14	RPA	0.1 copies/μL	40 min	Fluorescence or colorimetric (nanoprobe)	High fidelity for SNP detection and toxin recognition	Limited to ssDNA, complex sample preprocessing required	Aflatoxin B1 (toxin), HERC2	Harrington et al. (2018), Chen et al. (2023)

## 8 Summary and outlook

Traditional molecular diagnostic methods, such as PCR, rely on thermal cyclers and specialized laboratories. This requirement makes meeting the demand for POCT in remote areas and during outbreaks difficult. In contrast, IAT represents a revolutionary breakthrough, offering an isothermal reaction, minimal equipment requirements, and exceptional speed and efficiency. We focus on the principles and applications of core technologies such as NASBA, LAMP, RPA, and RAA. NASBA enables isothermal amplification of RNA targets through reverse transcriptase and RNA polymerase but is limited by temperature sensitivity and nonspecific amplification. LAMP uses a chain-substitution polymerase and multi-primer design to achieve rapid detection but has complex primer design and risks aerosol contamination. RPA and RAA simplify the process by combining recombinase and polymerase, which significantly increases assay speed, but they are limited by fragment length. In addition, IAT technologies combined with the CRISPR/Cas system, such as SHERLOCK and DETECTR, have achieved ultrahigh sensitivity and multiplex detection through highly specific detection and signal amplification by nucleases, demonstrating significant value in outbreaks of diseases such as Zika virus and COVID-19.

The concretization and expansion of future technological trajectories should prioritize the synergistic integration of innovation and practical implementation. First, the development of machine learning-driven intelligent platforms (e.g., AlphaFold-assisted enzyme engineering or CRISPR scan-optimized gRNA design platforms) is aimed at mitigating challenges related to nonspecific amplification in NASBA and off-target effects in CRISPR/Cas systematically (Moreno-Mateos et al., 2015; Cui et al., 2018; Peccati et al., 2023). By predicting enzymatic thermal stability and target binding affinities, these tools enable precise modulation of molecular interactions, thereby increasing reaction specificity. Additionally, machine learning algorithms can optimize primer design protocols, quantitatively assess primer ensemble performance, and minimize primer dimer formation and nonspecific amplification events, streamlining nucleic acid synthesis workflows (Dwivedi-Yu et al., 2023).

Second, advancements in miniaturized, automated pathogen detection systems leverage integrated technologies spanning isothermal amplification techniques, CRISPR-based signal transduction, and microfluidic chip architectures to create “sample-in, result-out” diagnostic devices (Qin et al., 2019; Didarian and Azar, 2025). Miniaturization enables seamless integration into mobile terminals or POCT kits, overcoming geographical barriers and democratizing access to diagnostics. End-to-end automation of sample preparation, reaction orchestration, and signal detection not only reduces operator-dependent errors but also expedites assay turnaround times, ensuring robust analytical sensitivity and specificity. The synergy of these technologies has accelerated the implementation of POCT, supporting public health emergency responses and equitable access to medical resources. In remote areas and during sudden outbreaks, such advancements will emerge as pivotal technological forces for safeguarding health and lives.

In the future, IAT will further advance molecular diagnostics, particularly in POCT scenarios in resource-limited areas. These technologies are uniquely fast, specific, and sensitive, enabling rapid and accurate pathogen detection. As they evolve, they hold the potential to transform diagnostics, personalized medicine, and environmental monitoring, making high-quality analytical tools accessible across diverse settings. IAT is poised to become a cornerstone of next-generation precision medicine, delivering efficient, portable, and cost-effective solutions for global infectious disease prevention and control.
